# Bibliometric and visual analysis of RAN methylation in cardiovascular disease

**DOI:** 10.3389/fcvm.2023.1110718

**Published:** 2023-03-30

**Authors:** Boce Song, Beili Xie, Mingwang Liu, Haohao Li, Dazhuo Shi, Fuhai Zhao

**Affiliations:** ^1^ Xiyuan Hospital, China Academy of Chinese Medical Sciences, Beijing, China; ^2^ Graduate School, Beijing University of Chinese Medicine, Beijing, China; ^3^ National Clinical Research Center for Chinese Medicine Cardiology, Beijing, China

**Keywords:** bibliometric analysis, RNA methylation, cardiovascular disease, visual analysis, citespace, VOSviewer

## Abstract

**Background:**

RNA methylation is associated with cardiovascular disease (CVD) occurrence and development. The purpose of this study is to visually analyze the results and research trends of global RNA methylation in CVD.

**Methods:**

Articles and reviews on RNA methylation in CVD published before 6 November 2022 were searched in the Web of Science Core Collection. Visual and statistical analysis was performed using CiteSpace 1.6.R4 advanced and VOSviewer 1.6.18.

**Results:**

There were 847 papers from 1,188 institutions and 63 countries/regions. Over approximately 30 years, there was a gradual increase in publications and citations on RNA methylation in CVD. America and China had the highest output (284 and 259 papers, respectively). Nine of the top 20 institutions that published articles were from China, among which Fudan University represented the most. The *International Journal of Molecular Sciences* was the journal with the most studies. *Nature* was the most co-cited journal. The most influential writers were Zhang and Wang from China and Mathiyalagan from the United States. After 2015, the primary keywords were cardiac development, heart, promoter methylation, RNA methylation, and N6-methyladenosine. Nuclear RNA, m^6^A methylation, inhibition, and myocardial infarction were the most common burst keywords from 2020 to the present.

**Conclusions:**

A bibliometric analysis reveals research hotspots and trends of RNA methylation in CVD. The regulatory mechanisms of RNA methylation related to CVD and the clinical application of their results, especially m^6^A methylation, are likely to be the focus of future research.

## 1. Introduction

Cardiovascular diseases (CVD) are the leading cause of death among noncommunicable diseases worldwide. Ischemic heart disease is the highest risk factor for premature death in women in more than half of countries and men in more than three-quarters of countries (1). Common risk factors for CVD include diabetes, hypertension, obesity, and smoking (2). CVD has been extensively studied at the genetic and epigenetic levels (3–5). Due to the close relationship between epigenetic regulation and environmental adaptation, the former has attracted substantial attention. As new insights are gained, epigenomics is emerging. Epigenetic transcriptomics does not cover all epigenetic regulation but focuses on RNA-level modification (6). RNA methylation accounts for the most significantly proportion.

RNA methylation refers to the methylation modification of RNA molecules at various positions, affecting gene expression. The molecules involved in RNA methylation are classified as Writers, Erasers, and Readers. Taking N^6^-methyladenosine (m^6^A) methylation as an example, for Writers, the methylation modification is “written” into RNA; that is, the methylation modification process of RNA is mediated (7). The most common molecules are methyltransferase-like 3 (METTL3) and METTL14. Studies have found that MettL3/14 rapidly locates disease gene loci and enriches m6A, promoting the RNA methylation process (8). “Erasers” of the RNA methylation modification signal mediate RNA demethylation modifications. The most common are obesity-associated protein (FTO) and AlkB homolog 5 (ALKBH5), which remove methyl groups on RNA (9). Readers “read” the information on RNA methylation modification and participate in downstream RNA translation, degradation, and other processes. For example, proteins with the YTH domain family proteins recognize and bind m^6^A in mRNA, reducing the half-life of mRNA and promoting its degradation (10). Depending on the methyl modification positions, it can be roughly divided into m^6^A, N^1^-methyladenosine (m^1^A), and 5-methylcytosine (m^5^C). m^6^A is the most abundant form, distributed near the stop codon and the 3′UTR region, affecting RNA pairing, changing the secondary structure of RNA, or being directly recognized by proteins, thereby regulating mRNA maturation, variable splicing, stability, and translation processes (11, 12). m^6^A is catalyzed by methyltransferase complex and removed by demethylated transferase ALKBH5 or FTO (13); m^1^A is a reversible apparent transcription modification removed by the RNA demethylated transferase ALKBH3. No m^1^A modification enzyme or modification recognition protein has been found. The expression abundance of m^1^A is low and distributed in the 5′UTR region of mRNA, which may be involved in regulating translation initiation (14). m^5^C is widely distributed in tRNA and rRNA, stabilizing the secondary structure of tRNA, affecting the anticodon loop conformation, and maintaining the fidelity of rRNA translation and other functions. m^5^C can be catalyzed by theNOP2/Sun RNA methyltransferase 2 or tRNA aspartic acid methyltransferase 1 and oxidized by the dioxygenase ten-eleven translocation to form 5-hydroxymethylcytosine. m^5^C does not affect base pairing but may enhance base stacking and the hydrophobic interaction between RNA and protein (15).

Research into RNA methylation increases yearly. Napoli et al. focused on clinical trials of sensitive epigenetic drugs for the treatment of heart failure (HF) and outlined the discovery of epigenetic biomarkers and cardiac remodeling characteristics (16). METTL3 silencing inhibits m^6^A RNA hypermethylation, inflammatory responses, and monocyte adhesion induced by oxidative stress. It reverses the changes in the expression levels of nucleotide-binding domain leucine-rich repeat pyrin domain-containing 1 and Kruppel-like factor 4 in endothelial cells under oxidative stress conditions (17). These findings suggest that RNA methylation regulates the development of CVD; nevertheless, the hotspots and trends in RNA methylation in CVD research have not been analyzed.

The bibliometric analysis enables qualitative and quantitative analysis of published literature and is used to analyze disciplinary knowledge structures and explore development trends (18), which traditional meta-scores and systematic reviews cannot achieve. Bibliometric analysis reveals hotspots and trends in research fields and analyzes the information from countries, journals, authors, and keywords of published articles (19). This analysis method has been widely used in various fields.

This study used bibliometric analysis with CiteSpace 1.6.R4 advanced, VOSviewer 1.6.18, and Microsoft Excel 2016 software to explore the research hotspots and trends of RNA methylation in CVD since 1991 and create a visual knowledge map. We hope to help scholars understand this field's scientific basis and research trends.

## 2. Methods

### 2.1. Data collection

**Figure 1:**
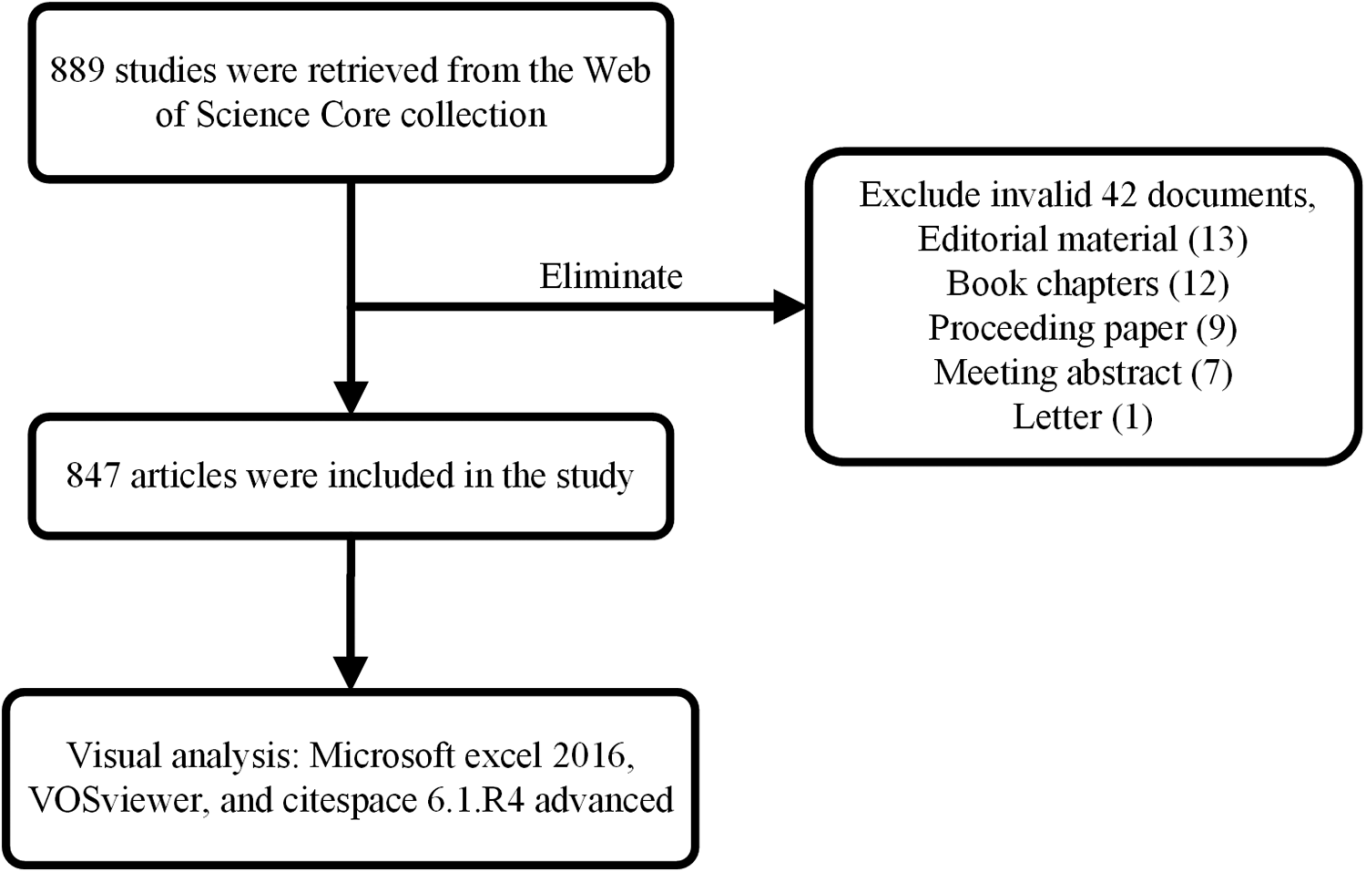
Flowchart of the study.

Articles were extracted from the Web of Science Core Collection (WoSCC) and were downloaded on November 06, 2022. The search terms were as follows: TS = (RNA methylation) AND (“circulation” OR “heart” OR “cardiovascular”), and the publication date: “1990-01-01” to “2022-11-06”, resulting in 889 records. We excluded invalid documents, including editorial material (13 articles), book chapters (12 articles), proceeding paper (nine articles), meeting abstract (seven articles), and letters (one article). Finally, 847 documents were included, and all records were exported and saved in plain text format. Figure 1 displays the flow chart.

### 2.2. Visual analytics

All valid documents retrieved from the WoSCC are exported into.txt files for visual analysis. We input the information into Microsoft Excel 2016 (Microsoft, United States) for the number of publications, the number of literature citations, and the analysis of research fields. CiteSpace 6.1.R4 advanced (CiteSpace for short, Drexel University, United States) and VOSviewer 1.6.18 (VOSviewer for short, the Centre for Science and Technology Studies, Holland) were used for literature clustering and visualization analysis. CiteSpace is a bibliometric analysis software developed by Chaomei Chen (20). It is a visual analytic tool for visualizing landmarks, critical paths, and emerging trends in a field of research based on relevant scholarly publications (21). We used CiteSpace to visualize the author and institutional collaboration, co-cited references and citation burst, keyword co-occurrence, and cluster and burst. The file was named starting with “download_” and imported into CiteSpace to remove duplicate files. There were no duplicate files. We imported the deduplicated files to CiteSpace for visual analysis. For institutional analysis, journal analysis, literature analysis, and author analysis, the parameter was set as “time slice 1 year” and “N = 50”; that is, the top 50 entries in each year were included in the analysis. The connection cable-cutting modes were pathfinder & pruning networks. To display image information more clearly, the time slice was set as two years in the keyword timeline view analysis, while other parameters remained unchanged. VOSviewer is an application for visual literature analysis, capable of coupling analysis of countries, journals, authors, citations, and more (22). We used VOSviwer to establish the item density of the countries. In the country analysis, the minimum number of articles per country was set at 1. In addition, the 2021 journal impact factors (IF) and JCR were obtained from the Web of Science.

## 3. Results

### 3.1. General analysis

#### 3.1.1. Number of publications and citations over the years

**Figure 2:**
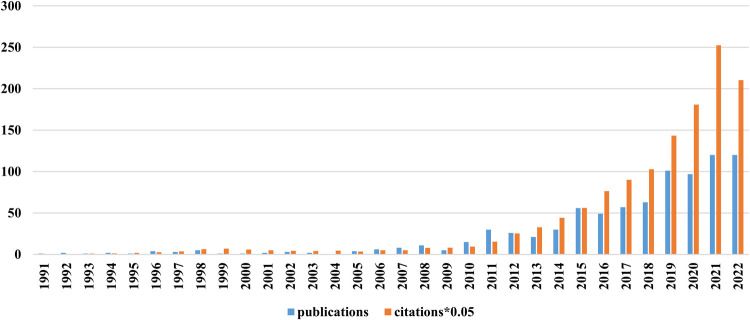
Annual publication and citation trends related to RNA methylation in CVD. Blue bars represent publications and yellow bars represent citations (*0.05).

The first RNA methylation article on CVD appeared in 1991. There was a small peak in 2008, and the number of published articles fell back in 2009. The number of published papers and citations increased steadily from 2010 to 2022. This finding indicates that RNA methylation has attracted the attention of researchers (Figure 2).

#### 3.1.2. Analysis of research areas

**Figure 3:**
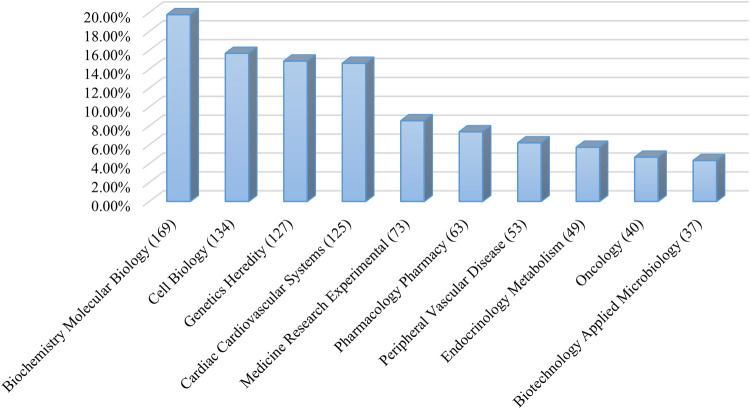
The top 10 research areas about RNA methylation in CVD.

The 847 articles covered 50 fields, including biochemistry and molecular biology (167 articles), cell biology (132 articles), genetics and heredity (124 articles), and biochemistry and cardiac cardiovascular systems (122 articles) were most common. Figure 3 shows the top ten research areas in RNA methylation in CVD.

### 3.2. Countries/regions and institutional analysis

#### 3.2.1. Analysis of countries/regions

847 articles were published in 1,188 institutions from 63 countries and regions. Table 1 displays the top 20 countries with the most significant number of publications. The top five countries/regions were the United States (23.87%), China (21.76%), Italy (6.13%), Germany (5.71%), and the United Kingdom (4.45%), accounting for 61.93% of the total publications.

**Table 1:** The top 20 authors and co-cited authors.

NO.	Author	Publication	Co-cited author	Co-cited
1	Francesco Paneni	10	Zhang Y	91
2	Claudio Napoli	9	Wang Y	90
3	Sarah Costantino	8	Mathiyalagan P	85
4	Gianluigi Condorelli	7	Wang X	82
5	Giuditta Benincasa	6	Meyer Kd	76
6	Lubo Zhang	6	Jones Pa	73
7	Thomas M Vondriska	6	Dorn Le	68
8	Simone Serio	5	Dominissini D	62
9	Leonardo Elia	5	Movassagh M	61
10	Paul H A Quax	5	Wang J	54
11	Jing Zhang	5	Jia Gf	53
12	Wei Sun	5	Zhang L	51
13	Jian Zhang	5	Liu Y	48
14	Shafeeq A Mohammed	5	Yang Y	47
15	Kun Wang	5	Berulava T	45
16	Roberto Papait	5	Li Y	45
17	Jing Chen	5	Song Hw	45
18	A Yael Nossent	5	Roundtree Ia	44
19	Philip A Marsden	5	Liu N	42
20	Daniel Levy	5	Zhou J	42

#### 3.2.2. Item density

**Figure 4:**
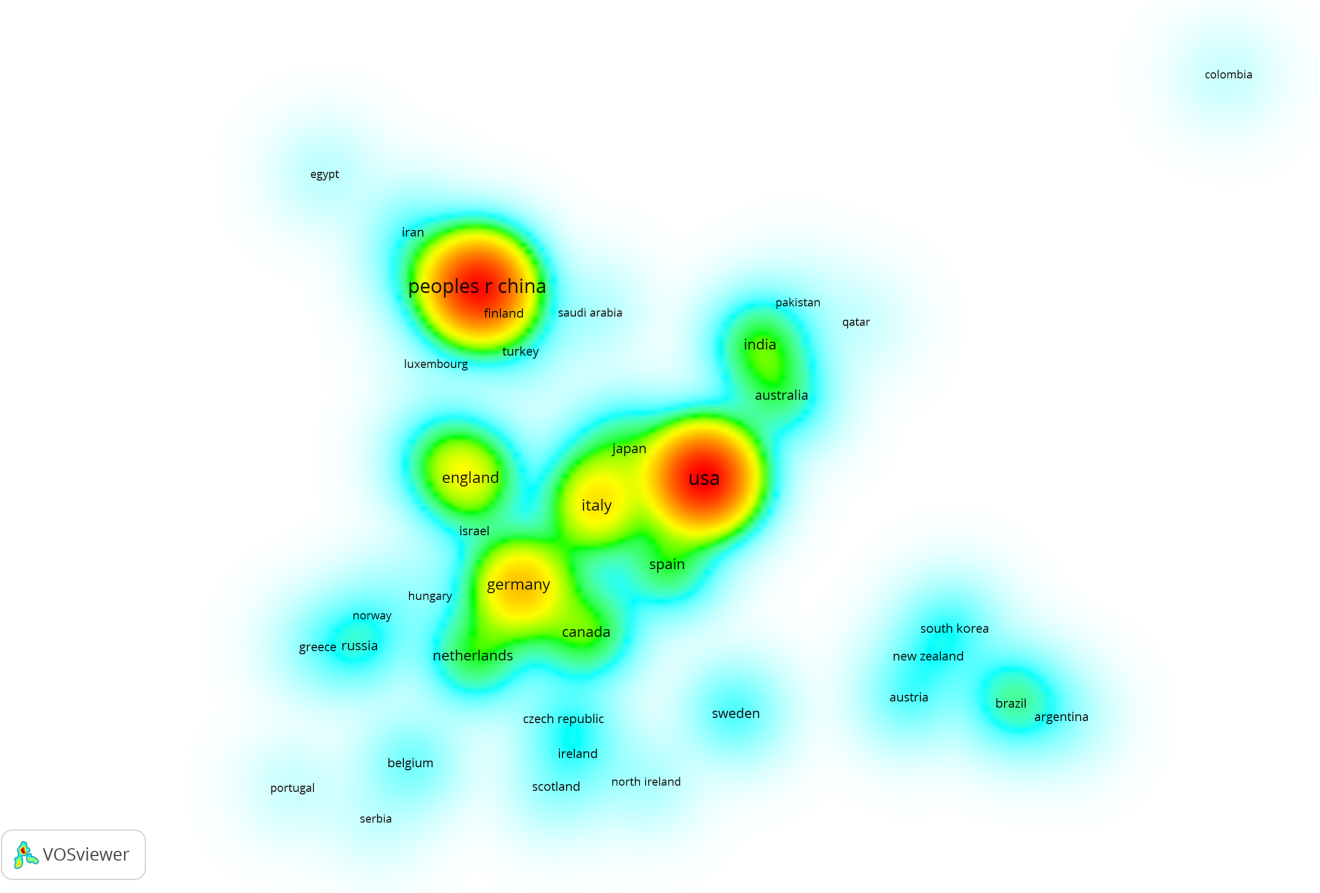
Item density of different countries in RNA methylation in CVD. The more items near a node, the higher the weight of adjacent items, and the closer the color of this point is to red.

Figure 4 shows that more items near a node mean a higher weight of adjacent items; red indicates the highest item density. The opposite is closer to blue. Countries with more than two articles were analyzed (Figure 4). As can be seen from the picture, there was close cooperation between the United States, Italy, and Japan. Although China has many publications, it shows few connections with other countries.

#### 3.2.3. Analysis of institutions

**Figure 5:**
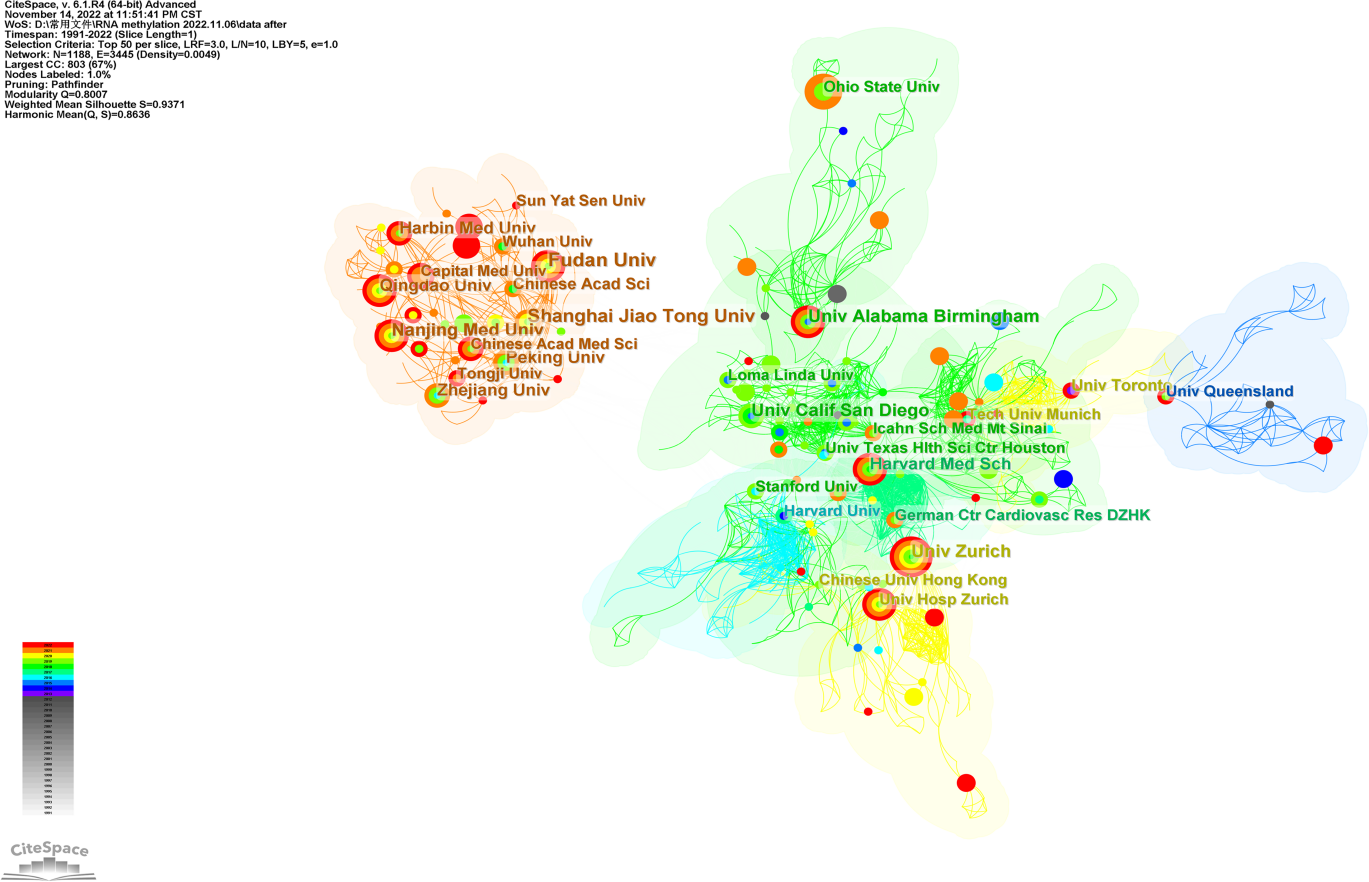
The visualization map of institutions involved in RNA methylation in CVD (*K* = 10). The size of nodes matches the publications of the institution. The links between nodes represent the institution collaborative relationships.

Table 2 shows the top 20 institutions in terms of publication volume among 1,188 research institutions, among which nine are Chinese research institutions. The top five were Fudan University (17 publications), Shanghai Jiao Tong University (15 publications), University of California, San Diego (14 publications), University of Zurich (14 publications), and Nanjing Medical University (14 publications). Figure 5 shows the top ten clusters (*K* = 10) established by CiteSpace analysis. Each cluster is displayed in a different color. Set parameters: The threshold is ten, and the node type is color ring history. The color of the node represents the year when the article was published. The reader should refer to the time color scale in the lower-left corner of the picture. The mechanisms at the ends of each line segment are cooperative relations. Modularity *Q* > 0.3 indicates significant modularity. Weighted Mean Silhouette *S* > 0.5 is reasonable clustering, and >0.7 means that the total cited clustering can clearly define each subdomain of clustering (23). Figure 5 contains 1,188 nodes and 3,445 lines, modularity *Q* = 0.8007, Weighted Mean Silhouette *S* = 0.9371, with high credibility. As shown in the figure, Fudan University, Nanjing Medical University, and Shanghai Jiao Tong University in China have close cooperation and are newly published; however, there was no evident cooperation relationship with institutions outside China. The University of California, San Diego, the Icahn School of Medicine at Mount Sinai, and the German Center for Cardiovascular Research work closely with Harvard Medical School but have not published recently.

**Table 2:** The top 20 institutions and countries/regions with the most publications.

NO.	Institutions	Count	Percentage	Countries/Regions	Count	Percentage
1	Fudan University	17	0.81%	United States	284	23.87%
2	Shanghai Jiao Tong University	15	0.71%	People's Republic of China	259	21.76%
3	University of California, San Diego	14	0.67%	Italy	73	6.13%
4	University of Zurich	14	0.67%	Germany	68	5.71%
5	Nanjing Medical University	14	0.67%	England	53	4.45%
6	The University of Alabama	14	0.67%	Canada	36	3.03%
7	University of California, Los Angeles	13	0.62%	India	34	2.86%
8	Qingdao University	12	0.57%	Netherlands	32	2.69%
9	Harbin Medical University	12	0.57%	Spain	31	2.61%
10	Peking University	12	0.57%	France	25	2.10%
11	Zhejiang University	12	0.57%	Australia	24	2.02%
12	Harvard Medical School	11	0.52%	Japan	23	1.93%
13	University of Toronto	10	0.48%	Switzerland	21	1.76%
14	Chinese Academy of Sciences	10	0.48%	Poland	13	1.09%
15	University of Milan	9	0.43%	Russia	13	1.09%
16	Chinese University of Hong Kong	9	0.43%	Sweden	12	1.01%
17	Technical University of Munich	9	0.43%	South Korea	11	0.92%
18	Stanford University	9	0.43%	Austria	10	0.84%
19	University Hospital Zurich	9	0.43%	Finland	10	0.84%
20	University of Campania Luigi Vanvitelli	9	0.43%	Brazil	10	0.84%

### 3.3. Journals and co-cited journals

#### 3.3.1. Dual-map overlay

**Figure 6:**
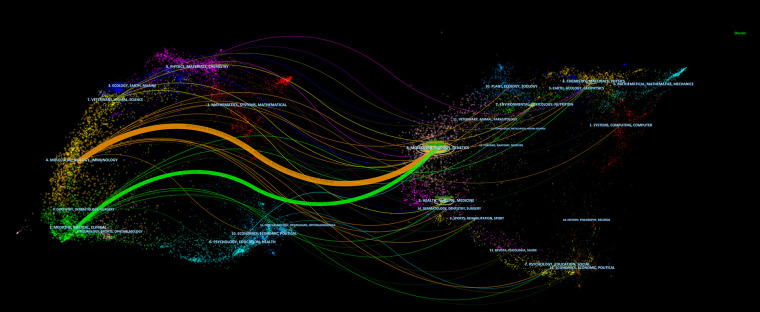
The dual-map overlay of journals on RNA methylation in CVD. Cited journals are on the left and cited journals are on the right. The link represents the cited path.

The dual-map overlay of journals shows the distribution of relationships between journals, citing journals on the left and cited journals on the right. The colored paths between them indicate the cited relationships. As shown in Figure 6, there are two primary citation paths, including one orange path and one green path. The two paths indicate that studies published in molecular/biology/immunology journals and medicine/medical/clinical journals cite from the studies in molecular/biology/immunology journals.

The dual-map overlay shows the citations between journals. As shown in Figure 6, cited journals are on the left, and cited journals are on the right. The colored curves between them indicate the relationship of citation. The orange and green curves represent the two primary reference paths, indicating that studies published in molecular/biology/immunology journals and medical/medical/clinical journals cited studies in molecular/biology/immunology journals.

#### 3.3.2. Journal analysis

Since 1991, 847 articles on RNA methylation have been published in 317 academic journals, among which 35 journals had an IF more significant than five. The top five journals were the *International Journal of Molecular Sciences* (27 publications), *Frontiers in Cardiovascular Medicine* (22 publications), *Circulation Research* (15 publications), *Frontiers in Cell and Developmental Biology* (15 publications), and *Clinical Epigenetics* (14 publications). The IF of Circulation was the highest (39.922). Among the top ten journals, six are in JCR Q1: *International Journal of Molecular Sciences* (IF = 6.208), *Circulation Research* (IF = 23.218), *Clinical Epigenetics* (IF = 7.28), *Circulation* (IF = 39.922), *Arteriosclerosis Thrombosis and Vascular Biology* (IF = 10.514), and *Frontiers in Genetics* (IF = 4.772). Six journals had an IF more significant than five (Table 3).

**Table 3:** The top 10 journals with the highest number of publications

NO.	Journal	Publications	*N*%	IF (2021)	CRJ (2021)
1	International Journal of Molecular Sciences	27	3.19%	6.208	Q1
2	Frontiers in Cardiovascular Medicine	22	2.60%	5.848	Q2
3	Circulation Research	15	1.77%	23.218	Q1
4	Frontiers in Cell and Developmental Biology	15	1.77%	6.081	Q2/Q1
5	Clinical Epigenetics	14	1.65%	7.28	Q1
6	Plos One	12	1.42%	3.752	Q2
7	Circulation	10	1.18%	39.922	Q1
8	Arteriosclerosis Thrombosis and Vascular Biology	9	1.06%	10.514	Q1
9	Frontiers in Genetics	9	1.06%	4.772	Q1
10	Journal of Molecular and Cellular Cardiology	9	1.06%	5.763	Q2

#### 3.3.3. Co-cited journals analysis

If papers are cited by one or more articles simultaneously, the two journals are said to have a co-citation relationship. The number of co-citations reflects the relevance of the literature in terms of content (24). CiteSpace was used for journal co-citation analysis, and there were 631 journals co-cited in this study. Thirty journals were cited more than 200 times, and nine were cited more than 400 times. As shown in Table 4, the top journals were *Nature* (620), *Proceedings of the National Academy of Sciences of the United States of America* (604), *Plos One* (549), *Cell* (547), *Science* (497), *Journal of Biological Chemistry* (472), *Nucleic Acids Research* (441), *Circulation* (440), *Circulation Research* (424), and *Nature Genetics* (380). Among the top ten cited journals, *Nature* had the highest IF of 69.504, followed by *Cell* (IF = 66.85), *Science* (IF = 63.832), and *Circulation* (IF = 29.69). Among the top 20 co-cited journals, 15 journals were CRJ Q1.

**Table 4:** The top 20 most co-cited journals

NO.	Co-cited journal	Count	Percentage	IF	CRJ (2021)	Year
1	Nature	620	3.79%	69.504	Q1	1992
2	Proceedings of the National Academy of Sciences of the United States of America	604	3.69%	12.779	Q1	1991
3	Plos One	549	3.36%	3.752	Q2	2009
4	Cell	547	3.35%	66.85	Q1	1991
5	Science	497	3.04%	63.832	Q1	1993
6	Journal of Biological Chemistry	472	2.89%	5.485	Q2	1991
7	Nucleic Acids Research	441	2.70%	19.16	Q1	1991
8	Circulation	440	2.69%	39.922	Q1	1996
9	Circulation Research	424	2.59%	23.218	Q1	1996
10	Nature Genetics	380	2.32%	41.376	Q1	1994
11	Nature Reviews Genetics	349	2.13%	59.924	Q1	2002
12	Journal of Clinical investigation	335	2.05%	19.477	Q1	1992
13	Molecular Cell	334	2.04%	19.328	Q1	2002
14	Nature Communications	327	2.00%	17.694	Q1	2015
15	Genes & Development	323	1.98%	12.89	Q1	1996
16	Scientific Reports	292	1.79%	4.997	Q2	2016
17	Molecular and Cellular Biology	288	1.76%	5.094	Q2	1991
18	New England Journal of Medicine	278	1.70%	176.082	Q1	1998
19	Biochemical and Biophysical Research Communications	278	1.70%	3.322	Q3	1992
20	Cardiovascular Research	263	1.61%	14.239	Q1	1996

### 3.4. Co-cited reference and reference burst detection

#### 3.4.1. Co-cited reference analysis

Co-citation refers to two or more papers being cited by one or more papers simultaneously (25). Table 5 displays the top ten co-cited references in which a co-citation occurred at least 20 times. “FTO-dependent N^6^-methyladenosine regulates cardiac function during remodeling and repair,” which was authored by Mathiyalagan and published in *Circulation*, was the most co-cited reference in RNA methylation in CVD (26), followed by the article entitled “The N^6^-methyladenosine mRNA methylase METTL3 controls cardiac homeostasis and hypertrophy”. Nine of the top ten most highly co-cited papers were experimental studies, and one was a review article.

**Table 5:** Articles co-cited more than 20 times.

NO.	First author	Title and document type	Journal	Co-citation
1	Mathiyalagan P	FTO-Dependent N^6^-Methyladenosine Regulates Cardiac Function During Remodeling and Repair (experimental study) (26)	Circulation	68
2	Dorn LE	The N^6^-Methyladenosine mRNA Methylase METTL3 Controls Cardiac Homeostasis and Hypertrophy (experimental study) (27)	Circulation	64
3	Song HW	METTL3 and ALKBH5 oppositely regulate m^6^A modification of TFEB mRNA, which dictates the fate of hypoxia/reoxygenation-treated cardiomyocytes (experimental study) (28)	Autophagy	44
4	Berulava T	Changes in m^6^A RNA methylation contribute to heart failure progression by modulating translation (experimental study) (29)	European Journal of Heart Failure	42
5	Roundtree IA	Dynamic RNA Modifications in Gene Expression Regulation (review) (30)	Cell	34
6	Kmietczyk V	m^6^A-mRNA methylation regulates cardiac gene expression and cellular growth (experimental study) (31)	Life Science Alliance	31
7	Zhao BX	Post-transcriptional gene regulation by mRNA modifications (review) (32)	Nature Reviews Molecular Cell Biology	28
8	Shi HL	YTHDF3 facilitates translation and decay of N^6^-methyladenosine-modified RNA (experimental study) (33)	Cell Research	25
9	Huang HL	Recognition of RNA N^6^-methyladenosine by IGF2BP proteins enhances mRNA stability and translation (experimental study) (34)	Nature Cell Biology	22
10	Jian D	METTL14 aggravates endothelial inflammation and atherosclerosis by increasing FOXO1 N6-methyladeosine modifications (experimental study) (35)	Theranostics	21

#### 3.4.2. Co-cited references burst detection

**Figure 7:**
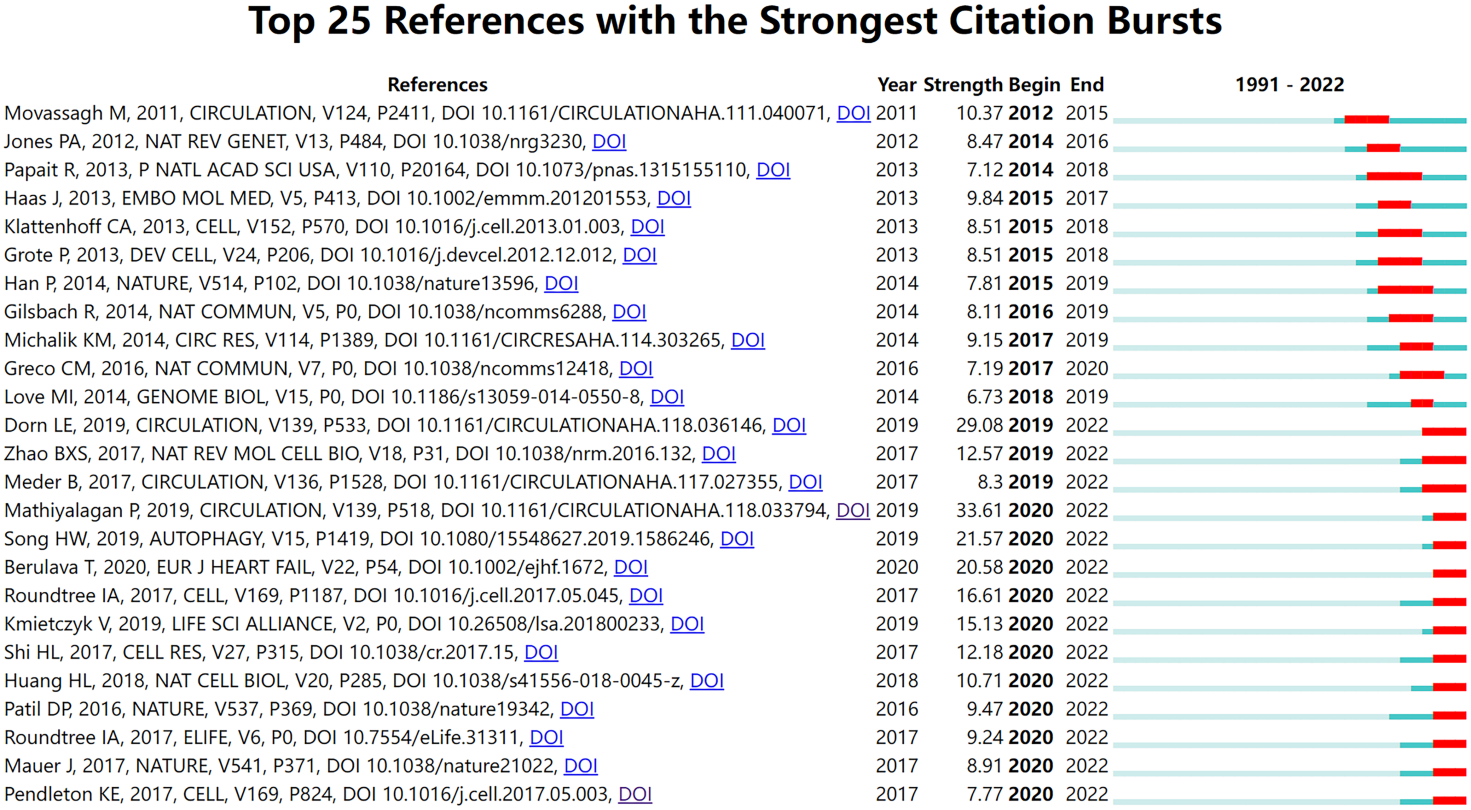
Top 25 references with the strongest citation bursts. The blue bar indicates that the reference has been published; the red bar means that the citation has exploded.

Burst detection identifies events that occur unusually frequently or suddenly during a period (36). Co-cited references burst detection can detect significant changes in citations during a specific period. It is used to find the decline or rise of a specific co-cited study. As shown in Figure 7, CiteSpace detected 25 references with the most citation bursts. The earliest reference with citation bursts began in 2012, entitled “Distinct epigenomic features in end-stage failing human hearts” by Movassagh et al. and published in *Circulation* (37). Since 2012, the number of studies cited has increased yearly. Among the 25 studies, 14 had bursts in the previous three years and continued until 2022. All articles published after 2019 were published in CRJ Q1.

### 3.5. Authors and co-cited authors

#### 3.5.1. Analysis of authors

5,116 authors were included in 847 studies. Table 1 shows the top 20 authors in the number of publications. Francesco Paneni published the most articles (*n* = 10), followed by Claudio Napoli (*n* = 9), Sarah Costantino (*n* = 8), and Gianluigi Condorelli (*n* = 7).

#### 3.5.2. Analysis of co-cited authors

**Figure 8:**
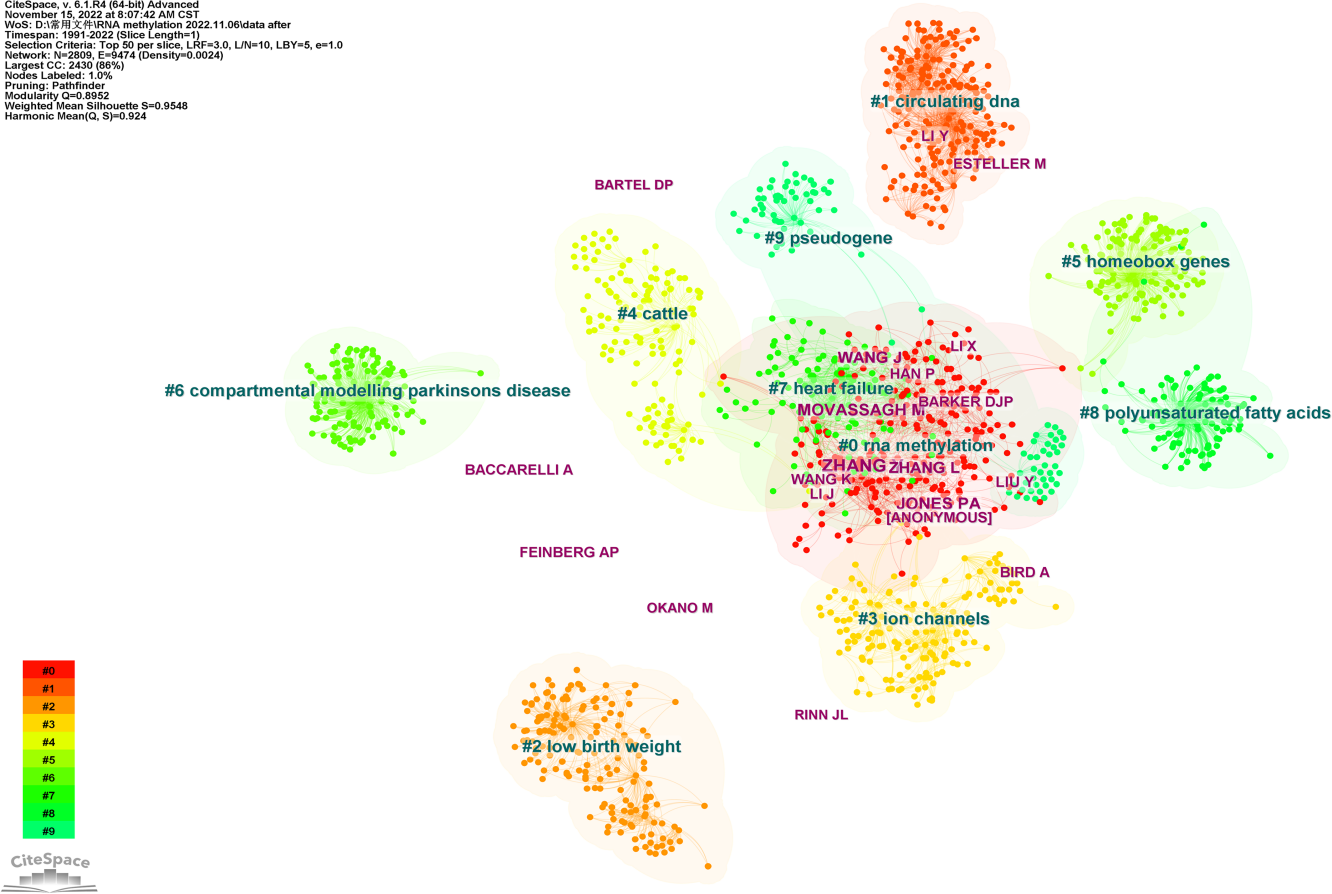
The visualization map of co-cited authors involved in RNA methylation in CVD (*K* = 10). The size of nodes matches the publications of the authors. Links between nodes represent co-citation relationships of authors.

If two (or more than one) authors were cited in one or more papers simultaneously, it is said that these two or more authors constitute a co-cited relationship. In this study, there were 2,809 co-cited authors, 12 of whom were cited more than 50 times, among which six were cited more than 70 times. Table 1 shows the top 20 authors with the most citations, of which the top five authors were Zhang (*n* = 91), Wang (*n* = 90), Mathiyalagan (*n* = 85), Wang (*n* = 82), and Meyer (*n* = 76). Figure 8 shows the top 10 clusters based on the keyword clustering for authors who co-cited over 30 times. The figure contains 2,809 nodes and 9,474 connections, Modularity *Q* = 0.8952, Weighted Mean Silhouette *S* = 0.8948. Each node represents an author, and more connections between nodes indicate closer author cooperation. The largest cluster in Figure 8 is 0#RNA methylation, containing 2,430 nodes, accounting for 86% of the total. Wang and Han, and Zhang Y and Zhang L showed close cooperation relationships.

### 3.6. Keyword co-occurrence and burst detection

#### 3.6.1. Keywords timeline view

**Figure 9:**
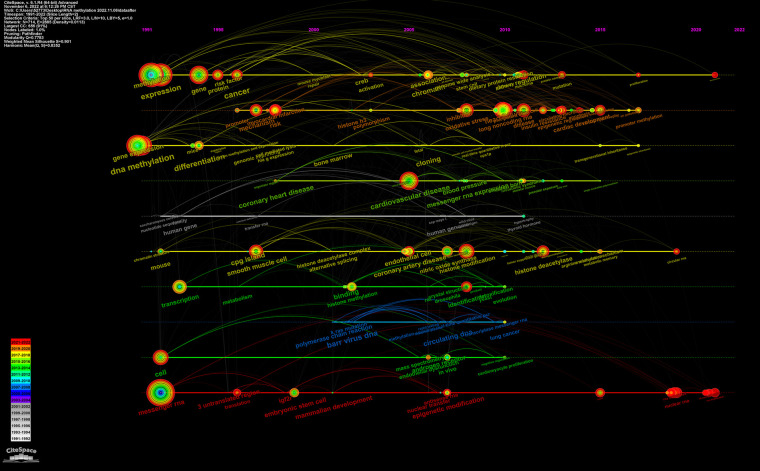
Timeline view of the CiteSpace visualization map relevant to RNA methylation in CVD. The horizontal lines of different colors represent the clusters formed by different keywords. The node on the horizontal line represents the keyword and its position represents the year in which the literature containing the keyword first appeared.

The keywords timeline view can reveal the period and research process of the development and evolution of various clusters. This research requires two years as a time slice to obtain the timeline view displaying keywords over time (Figure 9). Each node represents a keyword, and the node's size represents the cumulative use of the keyword from its appearance to the present. Keyword nodes appearing in different years show different tree ring colors. From 1991 to 2005, the top keywords were methylation, gene expression, DNA methylation, cell, messenger RNA, differentiation, transcription, and cgp island. From 1991 to 2005, chromatin, cardiovascular disease, ndothelial cells, coronary artery disease, oxidative stress, histone modification, and deacetylase were primary keywords. After 2015, the primary keywords were cardiac development, heart, promoter methylation, RNA methylation, and N^6^-methyladenosine.

#### 3.6.2. Keywords burst detection

**Figure 10:**
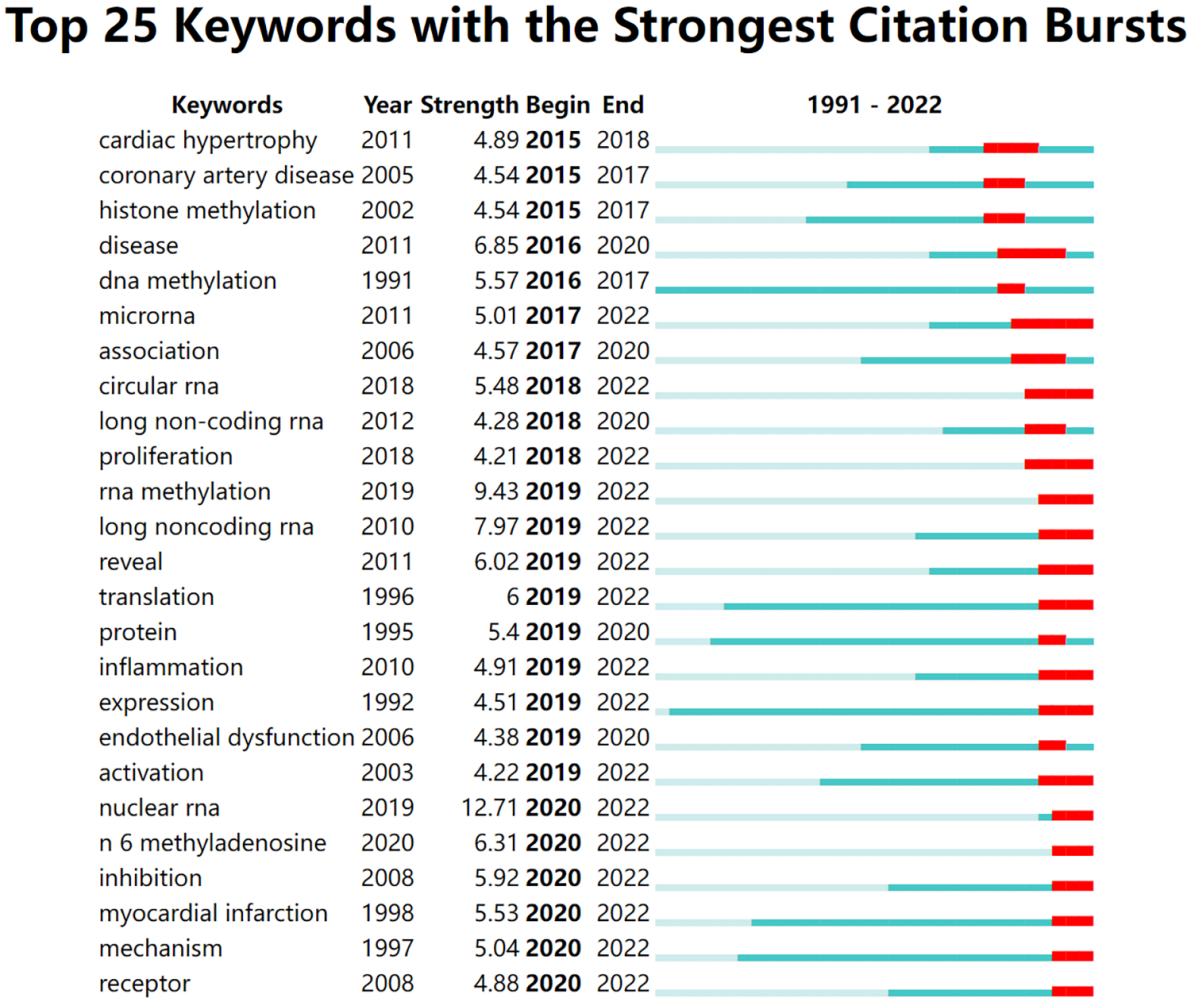
Top 25 keywords with the strongest citation bursts. The blue bar indicates that the keyword has been published; the red bar means that the keyword has burst.

In Figure 10, the green line segment's starting point represents the keyword's beginning and end. The red line segment represents the duration of keyword bursts. As we can see from the graph, there was no keyword burst before 2015. In 2016–2018, cardiac hypertrophy, coronary artery disease, DNA methylation, and histone methylation emerged as keywords. From 2016 to 2020, the number of keywords increased. These included inflammation, endothelial damage, RNA methylation, long noncoding RNA, proliferation, and others. Nuclear RNA, m^6^A methylation, inhibition, and myocardial infarction were new burst keywords from 2020 to the present.

According to the keywords burst detection, we can appreciate the rise of RNA methylation research from 2019. We identified articles after 2019. There were 83 articles with m^6^A as the topic, including 50 original studies, 13 articles with m^5^C as the topic (including four original studies), and only three review articles with m^1^A as the topic.

## 4. Discussion

### 4.1. General trend

The number of articles published in a field over the years can display the trend and popularity of research. Before 2010, the number of publications and citations related to RNA methylation in CVD was very small, with no papers in 2004 and only five in 2009. A turning point occurred in 2011, when 30 research papers were published, indicating that the field had captured the attention of researchers. Since then, the number of papers published has risen steadily. Since 2012, the number of citations of RNA methylation literature has increased rapidly, with a growth spurt from 2019 to 2021. Combined with the number of published articles and references cited, we infer that RNA methylation in CVD is rising rather than peaking. We expect that more articles will emerge in the next few years.

In national and institutional studies, the United States and China have an absolute advantage in the number of published articles; however, the institutions that publish articles are more scattered, and the number of published articles by a single research institution is not high. In contrast, though less than the United States in terms of the total number of articles published, China has the most among the top 20 institutions. This finding suggests that Chinese institutions such as Nanjing Medical University and Fudan University have a better research foundation and may make future breakthroughs in CVD RNA methylation.

In the journal analysis, the top ten journals were all in the CRJ Q1/Q2 area, suggesting that the outstanding journals have a high degree of recognition for the research results of RNA methylation. These results suggest that the study of RNA methylation in CVD has high credibility.

### 4.2. Research basis

The co-cited literature with high citations represents the research basis of a discipline. We interpreted the top five co-cited references.

The first co-cited paper was the experimental article published in *Circulation* by Mathiyalagan et al. (26). The functional roles of m^6^A and FTO in the heart and cardiomyocytes were investigated using clinical samples, pig and mouse models, and primary cardiomyocyte cultures. The authors found that the expression of the demethylation enzyme FTO was decreased in the HF model, and the increase of FTO expression in HF mice reduced the decline of systolic function induced by m^6^A. These findings suggest the functional importance of the FTO-dependent m^6^A methyl group in cardiac contraction in HF.

Second place went to Dorn et al. (27), published in *Circulation*. The authors showed that METTL3-mediated methylation of m^6^A mRNA is a dynamic modification. Enhanced m^6^A leads to compensatory myocardial hypertrophy, while inhibition of m^6^A leads to muscle cell remodeling and dysfunction, highlighting the importance of METTL3-mediated methylation of m^6^A mRNA for maintaining normal cardiac function.

Third place went to Song et al. (28) for an article published in *Autophagy*. This study found that METTL3 silencing enhanced autophagy and inhibited hypoxia/reoxygenation-induced apoptosis of cardiomyocytes. However, overexpression of METTL3 had the opposite effect, suggesting that METTL3 inhibits autophagy. The transcription factor EB activates its transcription by binding to the ALKBH5 promoter, while the inhibition of METTL3 downregulates the stability of mRNA and does not involve transcription inhibition. The essential function of RNA methylation in the bidirectional regulation of ischemic heart disease was revealed.

In fourth place was a study by Berulava et al. (29) published in the *European Journal of Heart Failure*. Changes in m^6^A RNA methylation outweighed changes in gene expression in mammalian models of HF. Moreover, changes in m^6^A methylation of mRNA affected the polymeric binding of corresponding transcripts in the heart, affecting protein balance in a transcription-independent manner.

Roundtree et al. (30) described RNA methylation with different modifications, including adenosine methylation and cytosine modifications. Adenosine methylation in studies includes m^6^A and m^1^A, while cytosine modifications include m^5^C and 5-hydroxymethylcytosine. This paper points out the technical difficulties of RNA methylation. Taking m^6^A methylation as an example, although m^6^A accounts for 0.2%–0.6% of all adenosine in mammalian mRNAs, only semi-quantitative detection can be performed within the transcriptome.

These studies explored the clinical, animal, and cellular levels. At present, there have been many studies on the methodology of RNA methylation. For example, XNA probe and CRISPR/Cas12a-powered flexible fluorescent and electrochemical dual-mode biosensor (38), mG-quant-seq (39), direct RNA sequencing (40), and other techniques provide technical support for the study of RNA methylation in CVD. We should also recognize the shortcomings of the research. The most prominent problems are the deviation of detection results due to low RNA methyl content (41, 42) and the lack of international standards for methylation studies. Although RNA methylation includes various types, current studies focus on the effects of m^6^A methylation on HF (43), ischemic cardiomyopathy (44, 45), and myocardial hypertrophy (46). Other diseases in CVD require further exploration.

### 4.3. Author analysis

Ten co-cited papers in the author's analysis were published by Francesco Paneni, assistant professor of cardiovascular epigenetics at the Schlieren Campus of the University of Zurich and team leader. A primary goal of Paneni's lab is to characterize the epigenetic mechanisms of the myocardium and vascular damage in the setting of cardiometabolic disturbances (47, 48).

Paneni's research team believes that epigenetic biomarkers should be regarded as the target of molecular intervention to treat diseases rather than blindly clinging to complex biological models of diseases (49). The aim is to apply epigenetic research to clinical applications rather than focusing on microscopic gene changes. One of these studies applied the central regulator SIRT1 to obese patients of different ages and found that SIRT1 affects the complex epigenetic control of p66sch or arginase II. NO availability and mitochondrial respiratory chain protein expression were affected to treat obesity and age-related early microvascular endothelial dysfunction (50). The team also reviewed drugs with known epigenetic effects and potential use in HF (51), including resveratrol (52), curcumin (53), and danshen (54).

The runner-up author, Claudio Napoli, published nine articles. Napoli's research on epigenetics is related to histones, microRNA, and DNA methylation, dedicated to the precise treatment of CVD and its high-risk pathogenic factors from the level of gene methylation (55–57). The Napoli group performed reduced-representation bisulfite sequencing and found that aortic endothelial cells exposed to high glucose had higher methylation differences of vascular endothelial growth factor (3.6 times), nitric oxide synthase 3 (1.6 times), and gene expression than normal aortic endothelial cells (58).

Third, Sarah Costantino suggested that studying cell-specific epigenetic information and developing chromatin modification drugs could provide a personalized risk assessment and treatment for patients with cardiometabolic disorders (51). The team examined the effects of hypoxia and hypertrophy stimulation on cardiomyocyte function and methylation levels and found that both resulted in epigenetic-driven upregulation of tenascin C and subsequent damage to cardiomyocyte energy metabolism, revealing the role of RNA methylation in the pathogenesis of CVD (59).

Gene-targeted therapy (60), precision medicine (61), and personalized therapy (62) were frequently used in co-cited literature. It is not difficult to see that the current research on RNA methylation in CVD is no longer limited to the simple mechanism study of disease but is committed to the clinical application of research results.

Co-cited author analysis can obtain high-influence authors in RNA methylation in CVD. We identified the authors with similar research topics, which authors created liaisons among topics, and which authors have multidisciplinary backgrounds or research fields with interdisciplinary attributes (63, 64). In the analysis of co-cited authors, Zhang, Wang, Mathiyalagan, and Wang were all cited more than 80 times. This finding suggests that their research results substantially influence this field.

### 4.4. Research hotspot and trend analysis

Using CiteSpace for burst detection analysis, we discovered the evolutionary trends of research from macro to micro, from single to diversified, reviewed the period when keywords become hot and predicted which keywords will continue the explosive trend in the future (65).

The keyword burst detection analysis showed no keyword emergence before 2015, suggesting that RNA methylation was an emerging field. In 2016–2018, investigators began to study the relationship between RNA methylation and specific types of CVD, including cardiac hypertrophy (66, 67); and coronary artery disease (68, 69), and began to explore mechanisms such as DNA methylation and histone methylation. From 2016 to 2020, RNA methylation-mediated molecular mechanisms, including inflammation and endothelial damage, attracted attention. Endothelial dysfunction and inflammatory infiltration are essential processes that promote the development of atherosclerosis. High blood lipids, smoking, high blood shear stress, and other factors lead to abnormal vascular endothelial function, release adhesion factors and chemokines, such that monocytes in blood migrate to the arterial subendothelial space, differentiate into macrophages to take up lipids, and convert into foam cells, promoting atherosclerosis (70). Studies found that FTO can promote the demethylation of m^6^A by yes-associated protein 1 and reduce apoptosis and inflammation of hypoxia/re-oxygenation-induced cardiomyocytes (71). Lysine methyltransferase Smyd1 reduces IL-6 expression by enhancing methylation of trimethylation of lysine-4 of histone-3 within the IL-6 promoter (72). After 2020, m^6^A RNA methylation, nuclear RNA, inhibition, and other microscopic precise regulation gradually attracted investigator attention (73, 74).

Co-citation burst detection reflects the research nodes in the development of a specific field and is suitable for investigating research hotspots and predicting development trends (75). There were four articles with a burst intensity >20 (Figure 7), all of which were experimental articles published in CRJ Q1 journals. An experimental article published by Mathiyalagan et al. in 2019 (strength = 34.78, *Circulation*) was also the highest co-citation. From clinical to animal to cell, from *in vitro* to *in vivo* research, this paper comprehensively and meticulously demonstrated the role of FTO de-m^6^A methylation. This article represents a turning point in research methods and content (26). The second, an experimental article by Dorn et al. published in 2019 (strength = 29.9, *Circulation*), inhibited m^6^A RNA methylation *in vitro* and *in vivo*, clarifying the importance of RNA methylation for maintaining cardiac homeostasis (27). The authors of the third paper, Song et al. (strength = 22.39, *Autophagy*), revealed a link between METTL3-ALKBH5 and autophagy, providing a scientific basis for the role of reversible mRNA m^6^A methylation in ischemic heart disease (28). A fourth, published in 2020 by Berulava et al. (strength = 21.37, *European Journal of Heart Failure*), found that m^6^A methylation is altered in cardiac hypertrophy and HF with corresponding protein abundance but independent of mRNA levels, revealing a novel transcription-independent translation regulatory mechanism (29).

In RNA methylation studies, m^6^A is the most common methylation modification in mRNA, with approximately 25% of mRNA carrying at least one m^6^A site (76), while m^5^C and m^1^A are found in tRNA and rRNA at relatively low levels (77, 78). m^6^A methylation is currently the focus of research on RNA methylation in CVD due to the differences in the content of different methylation types and the limited detection techniques.

Combined with citation bursts and keyword bursts, myocardial infarction, myocardial hypertrophy, and HF are current research hot spots. The exploration of methylation regulators related to these diseases and the clinical application of their results, especially the research related to m^6^A methylation, will become the goal of future research. The current RNA methylation detection methods are few and expensive, including liquid chromatography-mass spectrometry (79) and high-throughput sequencing (80). Dot blot represents a cost reduction for RNA methylation detection; however, the procedure is tedious and may result in false positives (81). Currently, the research on RNA methylation in the field of CVD is in its early stages, and optimizing the detection method of methylation to reduce the detection cost is a future direction of efforts.

## 5. Limitations

There are some shortcomings in this study. First, it was conducted on November 6, 2022, and covered all previous articles; however, some data may have been missed because the 2022 WoSCC database is still open to relevant documentation. Second, all data were downloaded from the WoSCC database, and studies not collected in the WoSCC were missed. WoSCC is the most used database in econometric analysis and contains the most information in the relevant core articles. Because the research data's quality may affect the knowledge graph's credibility, we chose WoSCC for this study. Other bibliometric studies also reported these limitations (82, 83).

## 6. Conclusion

We used CiteSpace and VOSviewer software visually to analyze RNA methylation in CVD and found that it has research value and application prospects in cardiovascular science. The leading countries studying this issue are the United States and China; however, other countries and institutions should strengthen cooperation. The increasing number of articles published in core international journals reflects the increasing emphasis of researchers in this field. The research focus of scholars shifted from basic to clinical research. Currently, research on RNA methylation in cardiovascular science focuses on the mechanism of ischemic heart disease, biomarkers, targeted therapy, and (especially) m^6^A methylation. These areas will be the focus of future research.

## Data Availability

The raw data supporting the conclusions of this article will be made available by the authors, without undue reservation.
